# *FXYD3* Expression Predicts Poor Prognosis in Renal Cell Carcinoma with Immunosuppressive Tumor Microenvironment

**DOI:** 10.3390/cancers14153596

**Published:** 2022-07-23

**Authors:** Satoru Yonekura, Kosuke Ueda

**Affiliations:** 1 Gustave Roussy Cancer Campus (GRCC), 94800 Villejuif, France; 2 Department of Urology, School of Medicine, Kurume University, Kurume 830-0011, Japan; ueda_kousuke@med.kurume-u.ac.jp

**Keywords:** renal cell carcinoma, biomarker, *FXYD3*, microenvironment

## Abstract

**Simple Summary:**

*FXYD3* belongs to the protein-coding gene family associated with Na+/K+-ATPase enzymes and chloride ion channels. Recently, the biological role of *FXYD3* has been reported in multiple cancers. Nevertheless, the prognostic value of *FXYD3* expression has been undiscovered in clear renal cell carcinoma (KIRC). In this study, we assessed the datasets from The Cancer Genome Atlas (TCGA) and Gene Expression Omnibus (GEO) dataset (GSE29609). We found the *FXYD3* high KIRC patients had distinct clinical characteristics, including hypoxia and poor overall survival. Furthermore, the algorithms discovered that *FXYD3* mRNA levels were associated with tumor purity, multiple types of the tumor infiltrating lymphocytes (TILs) and several genes related to T cell exhaustion. In conclusion, *FXYD3* predicts a poor prognosis associated with hypoxia, pro-tumor TILs, and T cell exhaustion in KIRC.

**Abstract:**

*FXYD3* is a protein-coding gene, belonging to the FXYD protein family associated with Na+/K+-ATPase enzymes and chloride ion channels. Accumulating evidence suggests the biological role of *FXYD3* in multiple cancers. However, the prognostic value of *FXYD3* expression in clear renal cell carcinoma (KIRC) is unclear. Therefore, we evaluated the clinical data with tumor-infiltrating lymphocytes (TILs) and immunoinhibitory gene expression data using The Cancer Genome Atlas (TCGA) and Gene Expression Omnibus (GEO) dataset (GSE29609). First, the *FXYD3* high KIRC patients had distinct clinical characteristics, including age, sex, disease stage, histological grade, and hypoxia-related gene expressions. Next, *FXYD3* gene expression was correlated with poor overall survival in both TCGA and GSE29609 cohorts. The ESTIMATE algorithm revealed that higher *FXYD3* mRNA levels were associated with increased infiltration of immune cells and tumor purity. Moreover, the *FXYD3* high KIRC tissue harbored increased TILs such as B cells, CD8+ T cells, and M1 macrophage, whereas NK cells and neutrophils were decreased. In addition, we showed *FXYD3* was co-expressed with several immunoinhibitory genes related to T cell exhaustion such as *LGALS9*, *CTLA4*, *BTLA*, *PDCD1*, and *LAG3*. In conclusion, *FXYD3* is an unfavorable prognostic biomarker associated with hypoxia, pro-tumor TILs, and T cell exhaustion.

## 1. Introduction

Renal cell carcinoma (RCC) is the most common type of kidney cancer in adults, of which clear cell RCC (KIRC) accounts for most cancer-related deaths [1]. Clinical challenges in advanced KIRC lie in managing a poor prognosis caused by resistance to radiotherapy and chemotherapy [2]. KIRC harbors an immunogenic tumor microenvironment (TME) containing various tumor-infiltrating T lymphocytes (TILs) [3]. Patients with advanced and metastatic RCC have been treated with molecularly targeted agent monotherapy such as tyrosine kinase inhibitors (TKIs). However, since the advent of immune checkpoint blockade (ICB), the treatment of RCC has reached a significant turning point. ICBs that target programmed cell death1 (PD-1), programmed cell death-ligand 1 (PD-L1), and cytotoxic T-lymphocyte-associated antigen 4 (CTLA-4) or ICB plus TKIs are now standard treatment options for RCC [4,5]. In the era of the ICB, an understanding of the immunogenic TME would be useful to find a new therapeutic strategy in KIRC management.

*FXYD3* is a protein-coding gene, belonging to the FXYD protein family in association with Na+/K+-ATPase enzymes and chloride ion channels [6]. *FXYD3* is expressed in various organs such as the liver, pancreas, stomach, colon, prostate, lungs, skeletal muscles, and kidneys [7]. In addition, evidence shows that *FXYD3* expression can be a poor prognostic biomarker in multiple solid tumors [8,9,10,11,12,13]. However, the prognostic role of *FXYD3* in KIRC has not been clarified so far. Therefore, we hypothesized *FXYD3* could predict the outcome in KIRC patients. Moreover, if *FXYD3* is associated with the TME of KIRC, *FXYD3* could be a potential target to modulate in order to increase the response of ICB in KIRC.

Herein, we investigated the prognostic value of *FXYD3* gene expressions in KIRC using The Cancer Genome Atlas (TCGA) datasets and Gene Expression Omnibus (GEO) datasets. Moreover, we investigated the tumor microenvironment (TME) by evaluating the estimated TILs [14] and immune-inhibitory gene expressions [15] in association with *FXYD3* expression.

## 2. Materials and Methods

### 2.1. Survival Analysis with FXYD3 mRNA Levels in the Public Database

Clinical data of patients with KIRC on TCGA datasets and GSE29609 [16] were obtained from cBioPortal (TCGA PanCancer Atlas; https://www.cbioportal.org/, accessed on 16 December 2020) [17,18], and GEO database, respectively. The patients with an upper 50% expression of *FXYD3* mRNA in each dataset were classified as *FXYD3* high, and the rest were classified as *FXYD3* low in all the following analyses. Kaplan-Meier curves were plotted for overall survival (OS). Data of tumor purity (ESTIMATE score), the level of stromal cells present (stroma score), and the infiltration level of immune cells (immunity score) based on expression data were obtained from ESTIMATE (https://bioinformatics.mdanderson.org/estimate/disease.html, accessed on 17 July 2022) [19]. *FXYD3* expression levels with tumor grade and disease stage were achieved on TISIDB [20]. The multivariate Cox regression analysis was used on Gene Expression Profiling Interactive Analysis 2 (GEPIA2), a web tool for comprehensive analyses with TIL data on TCGA [21,22], to evaluate the prognostic significance of *FXYD3* gene expression as a continuous variable with other parameters such as age, sex (male vs. female), and disease stage at initial diagnosis.

### 2.2. Estimated TIL Fraction

The TIL fraction data estimated using quanTIseq [14] was downloaded (http://timer.comp-genomics.org/, accessed on 17 July 2022). The composition of TILs was visualized by principal component analysis. The estimated TIL fractions between *FXYD3* high vs. low patients were compared by the Mann–Whitney test. A heatmap of TIL fractions estimated using quanTIseq [14] with clinical information was generated by the R package ‘ComplexHeatmap’. Each fraction of TILs was converted to a z-score. We performed hierarchical clustering to characterize the TILs, and the calculating methods of the distance for rows and columns were ‘canberra’ and ‘euclidean’, respectively. The clustering methods for rows and columns were ‘ward.D2’ and ‘complete’, respectively.

### 2.3. Gene Co-Expression Network Analysis

We used the R package ‘igraph’ to visualize the co-expression network of *FXYD3* and the immune-inhibitory genes [15]. Spearman’s correlation coefficients were used to generate the network graph. The nodes represented the genes and the edges corresponded to the interaction between genes. Subgraphs that show densely connected genes in the graph were created by calculating the leading non-negative eigenvector.

### 2.4. Statistical Analysis

For graphs and statistical analyses (principal component analysis, Mann-Whitney test), we used the R freeware (http://www.r-project.org, accessed on 17 July 2022) and GraphPad Prism (v. 9.4.0) software (GraphPad Software Inc., San Diego, CA, USA). All *p*-values were two-sided, and a *p*-value of ≤ 0.05 was considered statistically significant.

## 3. Results

### 3.1. Clinical Characteristics by FXYD3 Expression Level in KIRC Patients

Table 1 shows the clinical characteristics between *FXYD3* high and low patients (N = 255, *n* = 255, respectively). Significant differences of age, sex, disease stage, and histologic grade were detected. In the high *FXYD3* group, the median of age and the % of male patients were significantly higher. Moreover, the *FXYD3* high patients harbored more hypoxia-related gene expression by Buffa hypoxia score [23]. In contrast, there was no significant difference in mutation counts.

### 3.2. Prognostic Impact of FXYD3 Gene Expression in KIRC

**Figure 1:**
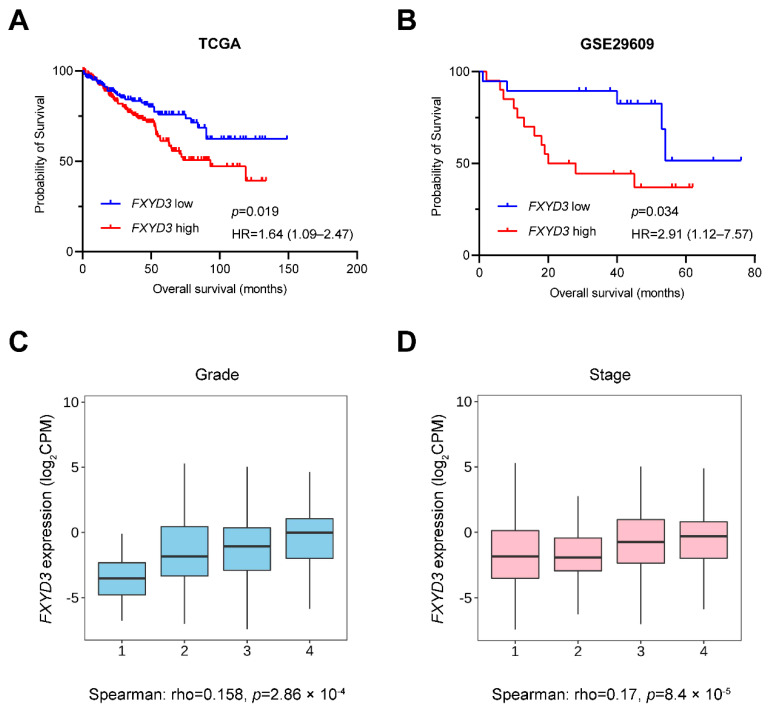
(**A**,**B**) Kaplan-Meier survival curves in *FXYD3* high vs. low patients with KIRC in TCGA (**A**) and GSE29609 (**B**), respectively. The outcome is overall survival. The log-rank test calculated *p*-value. HR, hazard ratio. (**C**,**D**) Box plots of *FXYD3* gene expression in each histological grade (**C**) and disease stage (**D**). KIRC, clear renal cell carcinoma. CPM, counts per million.

We investigated the OS using Kaplan-Meier curves in TCGA dataset (Figure 1A). Patients with higher *FXYD3* gene expressions had shorter OS on TCGA dataset (Hazard ratio (HR) (95% confidence interval (CI)): 1.64 (1.09–2.47), *p* = 0.019, Figure 1A). Similarly, in the other cohort (GSE29609 [16]), higher *FXYD3* gene expression was correlated with poor OS (HR (95% CI): 2.91 (1.12–7.57), *p* = 0.034, Figure 1B). Multivariate Cox regression analysis showed that higher *FXYD3* gene expression was significantly correlated with poor OS (HR (95% CI): 1.117 (1.013–1.232), *p* = 0.027, Table 2) on TCGA cohort. In addition, the patients with higher *FXYD3* expression had higher histologic grade of tumor and stage of disease (Spearman’s rho = 0.158, *p* = 2.86 × 10^−4^; Spearman’s rho = 0.17, *p* = 8.4 × 10^−5^, respectively, Figure 1C,D).

### 3.3. Infiltrating Immune Cells and FXYD3 mRNA Levels

**Figure 2:**
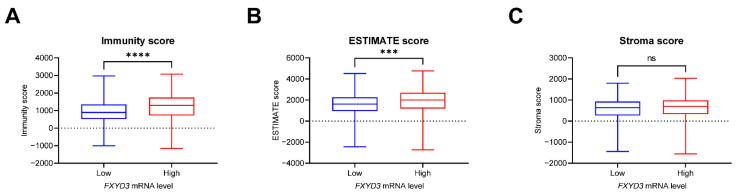
(**A**–**C**) Boxplots of immunity score (**A**), ESTIMATE score (**B**), and stroma score (**C**) between *FXYD3* expression low vs. high groups. Immunity score corresponds to the infiltration level of immune cells in tumor tissue. ESTIMATE score infers tumor purity based on expression data. Stroma score infers the level of stromal cells present. ***: *p* < 0.001 by the Mann–Whitney test. ****: *p* < 0.0001 by the Mann–Whitney test. ns, not significant.

**Figure 3:**
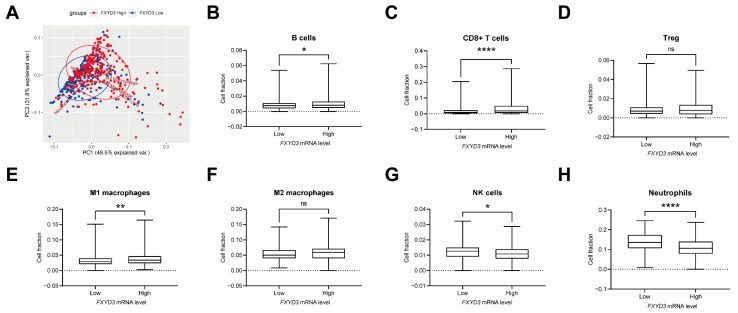
(**A**) Biplot of the principal component analysis of fractions of tumor-infiltrating lymphocytes (TILs) estimated by quanTIseq algorithm. Red dots correspond to *FXYD3* high, and blue ones correspond to *FXYD3* low patients’ data. (**B**–**H**) Boxplots of comparison of the estimated TILs fractions between low vs. high *FXYD3* groups: (**B**) B cells; (**C**) CD8+ T cells; (**D**) Regulatory T cells (Treg); (**E**) M1 macrophages; (**F**) M2 macrophages; (**G**) Natural killer (NK) cells; (H) Neutrophils. All statistical tests used in B-H were the Mann–Whitney test. *: *p* < 0.05. **: *p* < 0.01. ****: *p* < 0.0001. ns, not significant.

To understand the poor outcome in *FXYD3* high KIRC patients, we examined the characteristics of the TME between *FXYD3* high and low patients in terms of TILs, tumor purity, and abundance of stromal cells using the ESTIMATE algorithm [19]. We found *FXYD3* high KIRC patients had higher levels of infiltration of immune cells and tumor purity (Figure 2A,B) whereas there was no difference of stromal cells in the TME (Figure 2C). TILs are key factors in regulating tumor progression in KIRC [24]. Next, we compared the TILs estimated using the quanTIseq method [14] between *FXYD3* high and low groups. The TIL population composition did not strongly overlap based on *FXYD3* expression levels (Figure 3A). Next, the *FXYD3* high group had increased B cells, CD8+ T cells, and M1 macrophages, and decreased NK cells and neutrophils (Figure 3B,C,E,G,H). Regulatory T cells (Treg) and M2 macrophages were not significantly different (Figure 3D,F).

**Figure 4:**
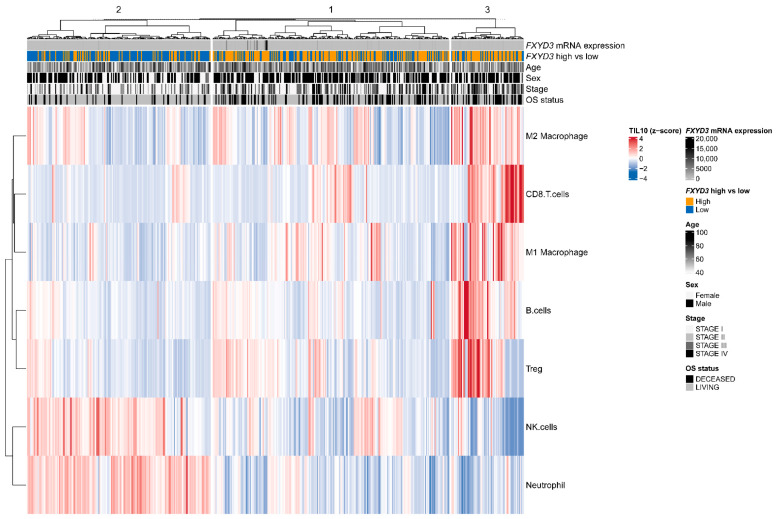
Heatmap of *FXYD3* mRNA levels, clinical information (age, sex, stage, overall survival (OS) status), and TIL fractions. Hierarchical clustering for rows and columns was performed. TIL fractions were estimated using the quanTIseq method. Treg, regulatory T cells. NK cells, natural killer cells.

The heatmap of TILs and *FXYD3* mRNA levels is shown in Figure 4. Three clusters were identified. In clusters 1 and 3, most patients were *FXYD3* high, while most of the cluster 2 patients had low *FXYD3* gene expression. The cluster 3 patients had high fractions of TILs, including M1/M2 macrophages, CD8+ T cells, B cells, and Treg, with fewer NK cells and neutrophils. The cluster 1 patients had similar TIL patterns to cluster 3, though all those TILs (i.e., M1/M2 macrophage, CD8+ T cells, B cells, and Treg) were relatively fewer than cluster 3. Contrary to clusters 1 and 3, the cluster 2 patients harbored increased NK cells and neutrophils and fewer M1/M2 macrophages, CD8+ T cells, B cells, and Treg.

### 3.4. Expression of Immunoinhibitory Genes and FXYD3 mRNA Levels

**Figure 5:**
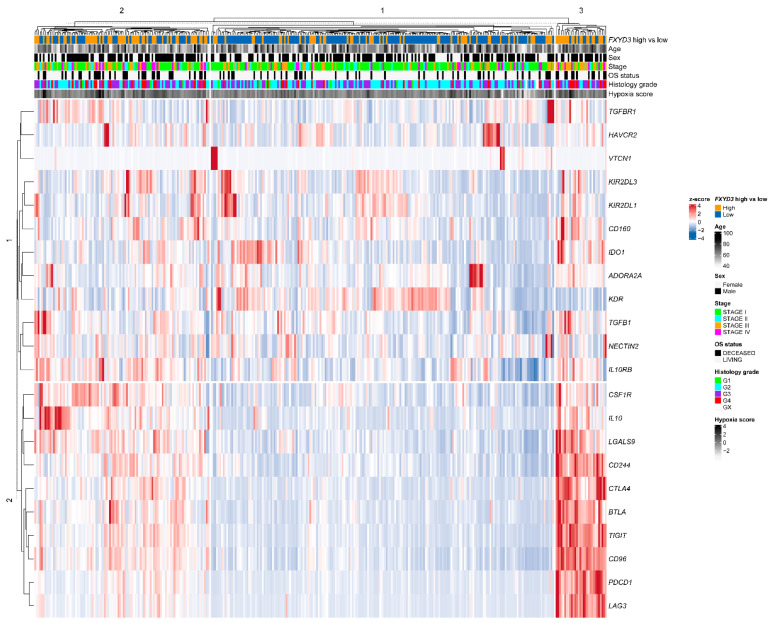
Heatmap of *FXYD3* mRNA levels, clinical information (age, sex, stage, overall survival [OS] status, histology grade, Buffa hypoxia score), and immunoinhibitory gene set. Hierarchical clustering for rows and columns was performed.

**Figure 6:**
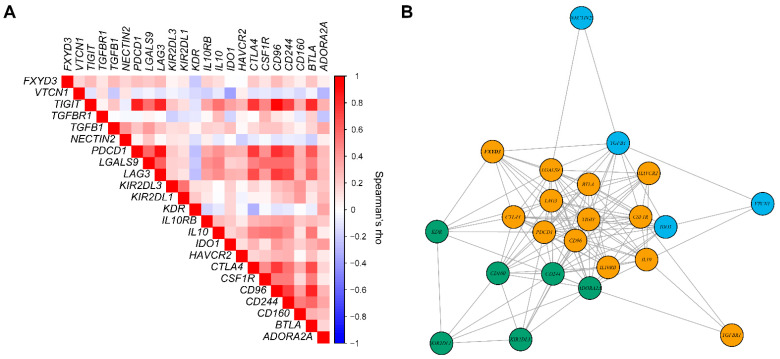
(**A**) Heatmap of correlation matrix of *FXYD3* mRNA levels with the immunoinhibitory gene set in KIRC patients. The colored scale shows the values of Spearman correlation coefficients (rho). (**B**) Gene interaction network of the genes in KIRC. The nodes represent the genes and the edges indicate the interaction between genes. Densely connected subgraphs within the graph are shown using different colored nodes by calculating the leading non-negative eigenvector.

To discover which immunosuppressive gene signals contribute to a worse prognosis in *FXYD3* high patients, we screened the expression of the immunoinhibitory gene set [15]. The heatmap of *FXYD3* mRNA levels and immunoinhibitory gene expression data is shown in Figure 5. Three clusters were identified. In column clusters 2 and 3, the majority of patients had higher *FXYD3* expression, while column cluster 1 was composed of more *FXYD3* low patients. The expression levels of row cluster 1 genes (*CD160*, *KIR2DL1*, *KIR2DL3*, *KDR*, *ADORA2A*, *IDO1*, *HAVCR2*, *NECTIN2*, *TGFB1*, *TGFBR1*, *VTCN1*, and *IL10RB*) were not distinctly different across all column clusters. In contrast, the row cluster 2 gene expressions (*CSF1R*, *IL10*, *LGALS9*, *CD244*, *CTLA4*, *BTLA*, *TIGIT*, *CD96*, *PDCD1*, and *LAG3*) were higher in column clusters 2 and 3, whereas column cluster 1 rarely expressed those genes. In addition, we evaluated the correlation of *FXYD3* gene expression with the immunosuppressive gene set in KIRC patients (Figure 6A). *FXYD3* had positive correlations with most of the gene set: *VTCN1*, *TIGIT*, *TGFBR1*, *TGFB1*, *NECTIN2*, *PDCD1*, *LGALS9*, *LAG3*, *IL10RB*, *IL10*, *CTLA4*, *CSF1R*, *CD96*, *CD244*, and *BTLA*. Only *KDR* was negatively correlated with *FXYD3*. Next, we performed a network analysis based on gene correlations to visualize the gene interactions (Figure 6B). Figure 6B shows gene correlation networks with Spearman’s rho values larger than 0.2. Three clusters were identified. Cluster 1 (yellow in Figure 6B) included *FXYD3*, *TIGIT*, *PDCD1*, *LGALS9*, *LAG3*, *CTLA4*, *CSF1R*, *CD96*, *BTLA*, *IL10RB*, *IL10*, *HAVCR2*, and *TGFBR1*. Cluster 2 (blue in Figure 6B) had *TGFB1*, *IDO1*, *NECTIN2*, and *VTCN1*. The cluster 3 (green in Figure 6B) genes were *KDR*, *ADORA2A*, *KIR2DL3*, *CD244*, *CD160*, and *KIR2DL1*. *FXYD3* had 9 edges and nodes. The network analysis identified the genes interacting closely with *FXYD3*, such as *TGFB1*, *LGALS9*, *LAG3*, *CTLA4*, *KDR*, *BTLA*, and *PDCD1*.

## 4. Discussion

The present study shows the *FXYD3* mRNA level is a prognostic factor in KIRC. In the TME of KIRC, we observed the different infiltration of multiple TILs associated with *FXYD3* expression levels. Moreover, KIRC patients with higher *FXYD3* gene expression had distinct expression patterns of immunoinhibitory genes, suggesting T cell exhaustion was associated with *FXYD3*.

FXYD families are expressed widely in mammalian tissues. The role of *FXYD3* in malignancy has been investigated in a variety of cancers, including esophageal squamous cell carcinoma [25], gastric cancer [26], endometrial cancer [8], pancreatic cancer [27], breast cancer [28,29,30], hepatocellular carcinoma [13], glioma [31], lung cancer [32], and colorectal carcinoma [10,11,12,33,34,35]. *FXYD3* expression in tumor tissue revealed by immunohistochemistry was an unfavorable prognostic marker in hepatocellular carcinoma [10] and colorectal cancer [10,12], though Jin et al. reported a contradictory prognostic role in colorectal cancer using TCGA database [11]. Strong *FXYD3* expression was observed in the infiltrative type in gastric [26] and colon cancer [12]. The in vitro experiments suggest *FXYD3* can be involved in tumor proliferation [27,28,32]. In total, *FXYD3* could be involved in tumor cell behavior contributing to poor prognosis in multiple cancers. Further studies using KIRC cell lines would be warranted to investigate the biological role of *FXYD3* in KIRC.

Our study provides a potential explanation for the poor prognosis in *FXYD3* high KIRC. First, increased hypoxia-related signaling was suggested in *FXYD3* high groups. Hypoxia is one of the hallmarks in various solid tumors with a critical association with tumor genetic instability and prognosis [36]. In KIRC, most tumors harbor somatic inactivation of both VHL alleles with loss of function of the VHL tumor suppressor protein (pVHL) [36]. The loss of function of pVHL leads to activation of hypoxia-inducible factor (HIF), leading to tumor progression [36]. The potential relevance of *FXYD3* to hypoxia-related genes should be explored using animal models in further studies. Second, we observed that the TIL pattern in *FXYD3* high patients was characteristic of an unfavorable TME containing increased CD8+ T cells [4,37] in KIRC. In the TME of KIRC, highly infiltrated CD8+ T cells paradoxically could not contribute to a better prognosis, likely due to exhaustion of CD8+ T cells [4,38]. Our results showed that disease stage and histological grade were correlated with increased *FXYD3* expression. Moreover, we found increased expression of multiple inhibitory genes involved in T cell exhaustion (e.g., *PDCD1*, *CTLA4, TIGIT, LAG3* [39]) in *FXYD3* high patients. The results of the network analysis also support the association of *FXYD3* with several genes related to T cell exhaustion [39,40] in KIRC. In contrast, the *FXYD3* low patients had increased NK cells and neutrophils with fewer T cell subsets such as CD8+ T cells or Treg. High NK cell infiltration in RCC is correlated with a better prognosis [41,42]. Our results suggest that the NK cells inside the TME could contribute to a better prognosis in *FXYD3* low patients. A higher neutrophil–lymphocyte ratio is prognostic of poor prognosis in RCC [43]. Neutrophils in the TME have subtypes: antitumor (N1) and pro-tumor (N2) phenotypes [44]. N2 neutrophils can be induced by exposure of the neutrophils to regulatory factors like TGF-β [44]. The present study showed the low expression of TGF-β in *FXYD3* low patients, suggesting that infiltrated neutrophils might not gain a pro-tumor phenotype. Taken together, *FXYD3* is associated with poor prognosis in KIRC in relation to pro-tumor TME, potentially with hypoxia-related signaling, less NK cells, and exhausted CD8+ T cells.

The acknowledged limitations are that the present study was based only on the public database. In addition, this study did not examine the association between *FXYD3* expression and the effects of antitumor agents, including ICBs. A preclinical model study would reveal the causative biological role of *FXYD3* signaling in KIRC. Further study is necessary to demonstrate the mechanisms involving *FXYD3*-related signaling pathways on poor prognosis.

## 5. Conclusions

The present study demonstrates that *FXYD3* is an unfavorable prognostic biomarker in KIRC with hypoxia, pro-tumor TILs, and multiple genes related to T cell exhaustion.

## Figures and Tables

**Table 1:** Subject characteristics by *FXYD3* expression levels.

Characteristics	*FXYD3* Low *n* = 255	*FXYD3* High *n* = 255	*p* ^†^
Age (median [range])	60.0 (29–90)	61.0 (32–90)	0.047
Sex			
Male	150	175	0.021
Female	105	80	
Stage			
I	146	108	0.0026
II	34	32	
III	71	108	
IV	4	7	
Histologic grade			
G1	12	1	< 0.0001
G2	121	93	
G3	95	105	
G4	21	54	
GX	4	1	
Buffa hypoxia score (median [range])	−1 (−33–35)	5 (−19–43)	< 0.001
Mutation count (median [range])	49 (8–426)	55 (10–591)	0.272

Continuous values are expressed as median (range). ^†^ Continuous and categorical variables were subjected to the Mann–Whitney U test and Chi-square test, respectively.

**Table 2:** Multivariate Cox regression analysis on overall survival in TCGA dataset.

Variable	HR	95% CI	*p*-Value
Age at diagnosis	1.033	1.018–1.048	<0.0001
Male (ref: Female)	0.903	0.656–1.244	0.532
Stage at initial diagnosis (ref: Stage 0/I)			
II	1.311	0.704–2.440	0.394
III	2.519	1.675–3.788	<0.0001
IV	7.081	4.818–10.41	<0.0001
*FXYD3* mRNA level	1.117	1.013–1.232	0.027

*n* = 530. TCGA, The Cancer Genome Atlas; HR, hazard ratio; CI, confidence interval; ref, reference.

## Data Availability

The authors downloaded the TCGA gene expression data (https://www.cbioportal.org/) and GSE29609 (https://www.ncbi.nlm.nih.gov/geo/).
